# Attitude towards deprescribing and its association with frailty and complexity of medication regimen: A survey of older inpatients in a district health board in New Zealand

**DOI:** 10.1186/s12877-023-03878-2

**Published:** 2023-03-23

**Authors:** Mohammed A Mohammed, Jeff Harrison, Aleksandra Milosavljevic, Amy Hai Yan Chan

**Affiliations:** School of Pharmacy, Faculty of Medical and Health Sciences, The University of Auckland, Auckland, New Zealand

**Keywords:** Attitudes toward deprescribing, Medication regimen complexity, Frailty, Older inpatients

## Abstract

**Background:**

Older inpatients, particularly those with frailty, have increased exposure to complex medication regimens. It is not known whether frailty and complexity of medication regimens influence attitudes toward deprescribing. This study aimed to investigate (1) older inpatients’ attitudes toward deprescribing; (2) if frailty and complexity of medication regimen influence attitudes and willingness to deprescribe - a relationship that has not been investigated in previous studies.

**Methods:**

In this cross-sectional study, older adults (≥ 65 years) recruited from general medicine and geriatric services in a New Zealand hospital completed the revised Patients’ Attitudes Towards Deprescribing (rPATD) questionnaire. Hospital frailty risk score (HFRS) was calculated using diagnostic codes and other relevant information present at the time of index hospital admission; higher scores indicate higher frailty risk. Medication regimen complexity was quantified using the medication regimen complexity index (MRCI); higher scores indicate greater complexity. Logistic regression analysis was used to identify predictors of attitudes and willingness to deprescribe.

**Results:**

A total of 222 patients were included in the study, the median age was 83 years and 63% were female. One in two patients reported feeling they were taking too many medications, and 1 in 5 considered their medications burdensome. Almost 3 in 4 (73%) wanted to be involved in decision-making about their medications, and 4 in 5 (84%) were willing to stop one or more of their medications if their prescriber said it was possible. Patients with higher MRCI had increased self-reported medication burden (adjusted odds ratio (AOR) 2.6, 95% CI 1.29, 5.29) and were more interested in being involved in decision-making about their medications (AOR 1.8, CI 0.99, 3.42) than those with lower MRCI. Patients with moderate HFRS had lower odds of willingness to deprescribe (AOR 0.45, CI 0.22,0.92) compared to the low-risk group. Female patients had a lower desire to be involved in decision-making. The oldest old age group( > 80 years) had lower self-reported medication burden and were less likely to want to try stopping their medications.

**Conclusion:**

Most older inpatients wanted to be involved in decision-making about their medications and were willing to stop one or more medications if proposed by their prescriber. Medication complexity and frailty status influence patients’ attitudes toward deprescribing and thus should be taken into consideration when making deprescribing decisions. Further research is needed to investigate the relationship between frailty and the complexity of medication regimens.

## Background

As people age, their treatment preferences and goals of care will change due to changes in physiologic function, increased prevalence of comorbidities, frailty, and use of multiple medications [1]. Thus, medications that were once considered appropriate may become unnecessary, and in some cases, even harmful resulting in poorer health outcomes [2]. Exposure to polypharmacy (use of ≥ 5 medications) [3, 4] and potentially inappropriate medications (PIMs), where the potential harms outweigh the benefits [5–7] is increasingly common in older adults. Inappropriate medication use may occur from starting a new inappropriate treatment or a reduction in benefits and/or an increase in risks associated with an existing treatment. It is estimated that between 19% and 88.5% of older adults have exposure to at least one PIM [8, 9]. PIMs use and polypharmacy can lead to negative consequences such as adverse drug events (ADEs), non-adherence, impaired cognitive and functional status, falls, hospitalisation, and mortality [10–13]. Globally, there has been a growing recognition of the increasing burden of PIMs and polypharmacy and the need to address associated risks and improve the health outcomes of older adults. One such method of addressing this is through deprescribing.

Deprescribing is ‘‘the clinically supervised process of tapering or stopping an inappropriate medication with the goal of managing polypharmacy and improving patient outcomes’’ [14, 15]. Deprescribing is a holistic process that needs patient and clinician involvement in shared decision-making to ensure informed decisions and achieve desired outcomes [16]. Several validated tools have been developed to aid healthcare providers in identifying and minimising inappropriate medication use and its consequences [17]. There have also been initiatives to develop guidelines to facilitate evidence-based decision-making in deprescribing [18–20]. As patient engagement is a core element of the deprescribing process, understanding patients’ lived experiences with medications [21] and their perspectives about deprescribing [22] is critical for the success of deprescribing interventions.

Previous international studies have shown that the majority of older inpatients feel that they are taking too many medications [23] and are willing to have one or more of their current medications deprescribed [24]. However, factors affecting attitudes and willingness to deprescribe such as the complexity of medication regimen in hospitalised older adults have not been well explored in previous studies. In addition, no study has explored attitudes toward deprescribing in older adults in New Zealand. Therefore, this study aimed to investigate (1) attitudes of older inpatients toward deprescribing and factors affecting their willingness to deprescribe; (2) if frailty and complexity of medication regimen influence attitudes and willingness toward deprescribing. This relationship has not been investigated in previous studies.

## Methods

### Study setting and population

This is a prospective cross-sectional study. The revised patient attitudes towards deprescribing (rPATD) questionnaire were administered to eligible patients admitted to Auckland District Health Board (ADHB) geriatrics and general medicines services between February 2020 and March 2022. Patients were eligible if they had at least one long-term condition, were taking at least one prescription medication on admission, were not cognitively impaired, and were able to complete the rPATD questionnaire in English. All inpatients aged ≥ 65 years admitted to geriatrics and general medicines services were consecutively screened by clinical pharmacists and nurses for eligibility based on inclusion criteria and information documented on electronic health records, and eligible patients were then referred to a research assistant to obtain consent and undertake the survey. Following the survey administration, we reviewed each participant’s electronic health records to collect sociodemographic (e.g., age, sex, ethnicity, socioeconomic deprivation) and clinical (e.g., comorbidities, history of readmission) and medication data. Detailed information about medications such as name, dose, dose frequency, route of administration, and the indication was collected for each medication. The New Zealand deprivation index (Dep2013) was used to evaluate socioeconomic deprivation and the scores were categorised as least deprived (scores 1–3), moderately deprived (scores 4–7), and most deprived (scores 8–10). Medical conditions were coded using the International Classification of Diseases 10th Revision-Australian Modification (ICD-10- AM). ICD-10 code was used to quantify comorbidity using the Charlson comorbidity index [25].

### Attitudes toward deprescribing measure

The rPATD is a 22-item questionnaire designed to measure patients’ attitudes toward deprescribing [22]. Participants were asked to self-complete all rPATD items. We then selected six rPATD items as a priori outcome of interest to assess five key areas: perceived burden, concerns about stopping, desire to involve in decision-making, satisfaction with medications, desire to try stopping one or more medications, and willingness to deprescribe. The selected items were: (1) ‘‘I feel that my medicines are a burden to me’’, (2) ‘‘I would be reluctant to stop a medicine that I had been taking for a long time’’, (3) ‘‘I like to be involved in making decisions about my medicines with my doctors’’, (4) ‘‘I am satisfied with my current medicines’’, (5) ‘‘I would like to try stopping one of my medicines to see how I feel without it’’ and (6) ‘‘If my doctor said it was possible, I would be willing to stop one or more of my regular medicines’’. For each domain, the mean scores were computed from the mean of individual items within the domain.

### Assessment of frailty

Hospital frailty risk score (HFRS) is a validated measure designed to assess the risk of frailty in hospitalised patients based on the number of accumulated deficits [26]; the higher the score, the higher risk of frailty for a patient. In this study, the HFRS of each patient was calculated using ICD-10 and other relevant information on the patient’s record at the time of index admission. Based on their HFRS, patients are categorised as no risk (0), low risk (1–5), intermediate risk (5–15), or high risk (> 15). In our study, there was no patients in the ‘no risk’ and ‘high-risk’ categry thus, HFRS was dichotomised into either low or intermediate risk.

### Assessment of medication regimen complexity

The medication regimen complexity index (MRCI) tool was used to determine the patient-level complexity of medication regimen [27]. The MRCI is a 65-item objective measure of the complexity of a medication regimen categorised into three sections: dosage forms, dosing frequency, and additional instructions for use (e.g. splitting tablets). The total MRCI is the sum of the scores of all three sections; higher scores indicate greater medication complexity. Hospitalised older adults are likely to be on complex medication regimens, which may predispose them to non-adherence and increased risk of readmission [28]. As there is no data on cut-off points to guide the categorisation of level of regimen complexity, we categorised the total score into tertiles as low, medium, and high MRCI.

### Statistical analysis

Participants’ characteristics and responses to rPATD were summarised using descriptive statistics. Categorical variables were reported using frequency (%) and continuous variables were presented as means (standard deviation [SD]) or medians and interquartile range as appropriate. To evaluate the association of rPATD items with covariates (e.g. MRCI, HFRS and sociodemographic factors), item responses were dichotomised to ‘‘Agree’’ (i.e. strongly agree and agree) and ‘‘Disagree’’ (i.e. strongly disagree, and disagree, unsure). The MRCI was further categorised into binary groups (low-medium vs. high MRCI). Logistic regression was used to examine the association of frailty and medication regimen complexity with attitudes toward deprescribing. The association of rPATD with MRCI and frailty was adjusted for covariates such as age, sex, number of medication and comorbidities (measured by Charlson comorbidity index). As there was some degree of collinearity between frailty and medication regimen complexity variables in our dataset, the association between rPATD and the two variables was analysed in a separate model. All two-sided P-values < 0.05 were statistically significant. Analyses were performed using SPSS software, version 28.

## Results

### Sociodemographic and clinical characteristics of the study population

Of 268 eligible participants approached, 222 completed the rPATD survey. The median (IQR) age was 83 (76–88) and 63% were female. The majority were of European ethnicity (85%), and 18% were living in the most deprived areas. The median (IQR) number of medicines and medication regimen complexity index were 10 (7–13) and 25 (16–35), respectively. Almost half of the patients had high medication regimen complexity, 53% and 37% had low and moderate risk for frailty, respectively. Almost 1 in 4 patients had a history of readmission (28 days) and 44% had a history of falls (Table 1).

**Table 1:** Sociodemographic and clinical characteristics of the study population (N = 222)

Patient characteristics	
Age in years, median (IQR)	83 (76–88)
Age > 80, n (%)	127 (57.2)
Sex (female), n (%)	141 (64.5)
Ethnicity, n (%)	
NZ European	188 (84.7)
Asian	17 (7.7)
Pacifika	11 (5)
Māori*	6 (2.7)
Socioeconomic deprivation**	
Least deprived (1–3), n (%)	84 (37.8)
Moderately deprived (4–7), n (%)	98 (44.1)
Most deprived (8–10), n (%)	40 (18)
Number of medications, median (IQR)	10 (7–13)
Polypharmacy (≥ 5 medicines), n (%)	199 (89.6)
No polypharmacy, n (%)	23 (10.4)
Medication regimen complexity index (MRCI), median (IQR)	25 (16–35)
Low MRCI, n (%)	53(23.9)
Medium MRCI, n (%)	61(27.5)
High MRCI, n (%)	108(48.6)
History of falls (28 days), n (%)	98 (44.1)
History of readmission (28 days), n (%)	54 (24.3)
Hospital frailty score, median (IQR)	3 (2–6)
Low-risk, n (%)	141 (63.1)
Intermediate risk, n (%)	81 (36.5)
Charlson comorbidity index (CCI), median (IQR)	1 (0–3)
CCI 0–2, n (%)	119 (53.6)
CCI ≥ 2, n (%)	103 (46.4)

*Māori = Indigenous people of New Zealand (Aotearoa), ** NZ socioeconomic deprivation index score, with a high category indicating more deprived and a lower category indicating less deprived areas.

### Attitudes toward deprescribing

Item and domain-level responses to the rPATD questionnaire are presented in Tables 2 and 3, respectively. Analysis of responses to the selected 6 items showed that 19.4% of the patients believe their medications are a burden to them, 30% were positive about stopping, 73% wanted to be involved in decision-making about their medications, and 83.8% were willing to stop one or more of their medications if their doctor said it was possible. Conversely, 91.4% were satisfied with their current medications (Table 2).

**Table 2:** Attitudes of older inpatients toward deprescribing (*N* = 222)

rPATD items	Strongly Agree & Agree	Unsure	Strongly Disagree & Disagree
I spend a lot of money on my medicines (B1)	35 (15.8%)	5 (2.3%)	182 (82.0%)
Taking my medicines every day is very inconvenient (B2)	43 (19.4%)	5 (2.3%)	174 (78.4%)
I feel that I am taking a large number of medicines (B3)	102 (46.2%)	11 (5.0%)	108 (48.9%)
I feel that my medicines are a burden to me (B4)	43 (19.4%)	7 (3.2%)	172 (77.5%)
Sometimes I think I take too many medicines (B5)	87 (39.2%)	10 (4.5%)	125 (56.3%)
I feel that I may be taking one or more medicines that I no longer need (A1)	60 (27.0%)	34 (15.3%)	128 (57.7%)
I would like to try stopping one of my medicines to see how I feel without it (A2)	67 (30.2%)	14 (6.3%)	141 (63.5%)
I would like my doctor to reduce the dose of one or more of my medicines (A3)	57 (25.7%)	33 (14.9%)	132 (59.5%)
I think one or more of my medicines may not be working (A4)	55 (24.8%)	59 (26.6%)	108 (48.6%)
I believe one or more of my medicines may be currently giving me side effects (A5)	80 (36.0%)	21 (9.5%)	121 (54.5%)
I would be reluctant to stop a medicine that I had been taking for a long time (C1)	104 (46.8%)	22 (9.9%)	96 (43.2%)
If one of my medicines was stopped, I would be worried about missing out on future benefits (C2)	83 (37.4%)	23 (10.4%)	116 (52.3%)
I get stressed whenever changes are made to my medicines (C3)	50 (22.5%)	9 (4.1%)	163 (73.4%)
If my doctor recommended stopping a medicine, I would feel that he/she was giving up on me (C4)	27 (12.2%)	13 (5.9%)	182 (82.0%)
I have had a bad experience when stopping a medicine before (C5)	45 (20.3%)	14 (6.3%)	163 (73.4%)
I have a good understanding of the reasons I was prescribed each of my medicines (I1)	187 (84.2%)	8 (3.6%)	27 (12.2%)
I know exactly what medicines I am currently taking, and/or I keep an up-to-date list of my medicines (I2)	176 (79.3%)	11 (5.0%)	35 (15.8%)
I like to know as much as possible about my medicines (I3)	174 (78.4%)	8 (3.6%)	40 (18.0%)
I like to be involved in making decisions about my medicines with my doctors (I4)	162 (73.0%)	5 (2.3%)	55 (24.8%)
I always ask my doctor, pharmacist or other healthcare professional if there is something, I don’t understand about my medicines I5)	191 (86.0%)	4 (1.8%)	27 (12.2%)
If my doctor said it was possible, I would be willing to stop one or more of my regular medicines (G1)	186 (83.8%)	21 (9.5%)	15 (6.8%)
Overall, I am satisfied with my current medicines (G2)	203 (91.4%)	6 (2.7%)	13 (5.9%)

Aggregated domain level responses to the rPATD tool showed that almost one in five (18%) patients reported high perceived burden, about one in four (23.4%) endorsed appropriateness of deprescribing and one in seven (15.3%) had concerns about stopping medications. Conversely, most participants had a desire to be involved in the decision-making about their medications (81.5%) and were willing to have their medications deprescribed (83.8%) (Table 3).

**Table 3:** Domain-level responses to patients’ attitudes toward the deprescribing questionnaire (*N* = 222)

rPATD domain	Mean (SD)	Agree n (%)	Disagree n (%)
Burden	2.6(0.8)	40 (18)	182(81.9)
Appropriateness	2.7 (0.8)	52(23.4)	170(76.6)
Concerns	2.5(0.8)	34(15.3)	188(84.7)
Involvement	3.9(0.7)	181(81.5)	41(18.5)
Satisfaction with medicines	1.2(0.6)	203 (91.4)	19(8.6)
Willingness to deprescribe	1.1(0.5)	186(83.8)	36(16.2)

### Association of the complexity of medication regimen and attitudes toward deprescribing

In univariate analysis, a higher MRCI score was associated with increased self-reported medication burden (Crude OR (COR) 2.6, 95% CI 1.29, 5.29) and a greater desire to be involved in therapeutic decision-making (COR 1.8, CI 0.99, 3.42). Conversely, a higher MRCI score was associated with greater concern about stopping medications (COR 1.72, CI 1.01, 2.92). After adjusting for covariates (age, sex, number of medicines, and comorbidity), a higher MRCI score was associated with higher self-reported perceived burden (AOR 2.79, CI 1.02, 7.57). The multivariable analysis also showed that the oldest old (> 80 years) group had a lower desire to be involved in therapeutic decision-making (AOR 0.42, CI 0.21,0.82) and was less likely to want to try stopping their medications (AOR 0.39, CI 0.21,0.72) compared to 65–80 years old (Table 4).

**Table 4:** Multivariable analysis of the association of complexity of medication regimen and attitudes toward deprescribing (N = 222)

Variables	Willingness to stop	Wanting to stop	Perceived burden	Concerns about stopping	Involvement in decision	Satisfaction with medicines
	AOR 95%CI	AOR 95%CI	AOR 95%CI	AOR 95%CI	AOR 95%CI	AOR 95%CI
MRCI (ref: low MRCI)	1.31 (0.46, 3.69)	1.06 (0.45,2.53)	**2.79(1.02,7.57)**	1.71(0.78,3.71)	1.93(0.78,4.73)	1.39(0.36,5.45)
Age (ref: ≤ 80)	1.33 (0.64,2.76)	**0.39 (0.21,0.72)**	1.18(0.59,2.33)	0.7(0.40,1.22)	**0.42(0.21,0.82)**	0.98(0.37,2.61)
Sex (ref: male)	0.72(0.33,1.56)	0.59(0.32,1.01)	1.19 (0.57,2.48)	1.29(0.73,2.28)	0.84(0.44,1.60)	0.99(0.37,2.69)
Number of medicines*	0.72(0.92,1.12)	1.06 (0.98,1.15)	0.99 (0.91,1.09)	0.99(0.92,1.07)	0.87(0.99,1.07)	0.99(0.88,1.13)
CCI*	1.02(0.82,1.23)	1.06 (0.90,1.25)	0.92 (0.75,1.13)	1.07(0.92,1.23)	0.98(0.83,1.17)	0.93(0.72,1.19)

CCI- Charlson comorbidity index, MRCI- medication regimen complexity index, AOR- adjusted odds ratio, CI-confidence interval, bold values indicate variables with statistical significance at P-value < 0.05, * number of medicines continuous and *CCI continuous.

### Association of frailty and attitudes toward deprescribing

In multivariable analysis, an increase in frailty risk score was associated with lower odds of willingness to deprescribe (AOR 0.45, CI 0.21, 0.98) whilst increased number of medications was associated with a higher desire to try stopping one or more medications (AOR 1.06, CI 1.00, 1.23). History of falls was associated with lower odds of self-reported burden (AOR 0.46, CI 0.21, 0.98). Female patients reported a lower desire to be involved in decision-making (AOR 0.44 CI 0.23, 0.86) compared to male patients, and the oldest old age group had lower perceived burden (AOR 0.46, CI 0.22, 0.93) and were less likely to try stopping their medications to see how they feel without them (AOR 0.38, CI 0.20, 0.69) (Table 5).

**Table 5:** Multivariable analysis of the association of frailty risk and attitudes toward deprescribing (N = 222)

Variables	Willingness to stop	Wanting to stop	Perceived burden	Concerns about stopping	Involvement in decision	Satisfaction with medicines
	AOR 95%CI	AOR 95%CI	AOR 95%CI	AOR 95%CI	AOR 95%CI	AOR 95%CI
HRFS (ref: low risk)	**0.45 (0.21, 0.98)**	1.35 (0.69,2.62)	1.59 (0.74,3.42)	1.29(0.72,2.34)	0.80(0.42,1.54)	1.05(0.37,2.98)
Age (ref: ≤ 80)	1.53 (0.72,3.23)	**0.38 (0.20,0.69)**	**0.46 (0.22,0.93)**	0.68(0.39,1.19)	0.98(0.37,2.61)	1.45(0.55,3.79)
Sex (ref: male)	0.85(0.37,1.94)	0.58(0.30,1.09)	1.39 (0.65,2.98)	1.32(0.74,2.36)	**0.44(0.23,0.86)**	0.44(0.23,0.86)
Number of medicines*	0.99(0.93,1.08)	**1.06 (1.00,1.23)**	0.92 (0.75,1.23)	1.03(0.98,1.09)	1.04(0.98,1.11)	0.97(0.89,1.01)
CCI*	1.04(0.84,1.29)	1.05 (0.89,1.24)	0.92 (0.75,1.13)	1.07(0.92,1.24)	0.99(0.84,1.19)	0.93(0.72,1.19)
History of falls	0.72(0.33,1.57)	1.01 (0.53,1.93)	**0.46 (0.21,0.98)**	0.87(0.49,1.54)	1.02(0.54,1.94)	0.81(0.29,2.24)

CCI- Charlson comorbidity index, MRCI- medication regimen complexity index, AOR- adjusted odds ratio, CI-confidence interval, bold values indicate variables with statistical significance at P-value < 0.05, * number of medicines continuous and *CCI continuous.

## Discussion

This is the first inpatient survey that investigated attitudes toward deprescribing in relation to medication regimen complexity and hospital frailty risk score. The study provides insights into factors associated with attitudes toward deprescribing in older inpatients in New Zealand. We found that four in five older inpatients had low perceived medication burden and 3 in 4 wanted to be involved in decision-making about their medicines. Despite reporting overall satisfaction with their current medications, most patients had positive views about the appropriateness of deprescribing and were willing to have one or more of their medications stopped if their doctor said it was possible. Increased number of medications, the complexity of medication regimens and higher frailty risk score, oldest old age (> 80 years), female sex, and history of falls were key factors found to have a significant association with patients’ attitudes and willingness toward deprescribing.

Our finding that most participants were overall satisfied with their medications, and many were willing to consider deprescribing, is consistent with previous studies conducted in the community and inpatient settings in Europe and Australia [24, 29–32]. Despite high satisfaction with medications (91%), the high level of willingness to deprescribe (84%) in our study may be due to the low perceived medication burden (18%) and high level of multimorbidity (46% had a Charlson comorbidity index of ≥ 2) present in the study population. This could also be related to greater endorsement of the appropriateness of medicines observed in the study population.

Consistent with previous community based studies, participants in our study reported a greater desire to be involved in decision-making [30]. This contrasts a study conducted in inpatient setting in the UK, where participants had a lower desire to be involved in decision-making [32]. Patients’ desire to be involved in decision-making and willingness to consider deprescribing should be recognised by clinicians as a catalyst to initiate deprescribing in the hospital. However, successful implementation of deprescribing in the acute setting requires commitment of hospitals to establish multidisciplinary collaborative deprescribing services to improve in-hospital and post-discharge outcomes.

The average number of medications in our study population was 10, and 89.6% of the cohort had polypharmacy (≥ 5 medications) indicating a group with a potentially high disease burden. Patients with higher medication regimen complexity scores reported higher perceived burden and those with an increased number of medications had a strong desire to try stopping medications. Patients with such characteristics are more likely to be interested in being involved in shared-decision making about their medications and consider deprescribing appropriate, therefore, should be a priority group for medication reviews and deprescribing in hospitals. There is conflicting evidence regarding the association of the number of medications with willingness to deprescribe. Some studies reported a significant association [33, 34] similar to our findings whilst others have shown no significant association [30, 31, 35, 36]. This difference might be due to differences in the population characteristics. In some of these studies, patients were recruited from community pharmacies and GP practices and, those patients may have had repeat visits to their regular pharmacy or GP practice and their medications reviewed on a regular basis. Patients may be less willing to have their medications withdrawn if they perceive they are on an optimal treatment. No previous studies have comprehensively explored the relationship between the complexity of the medication regimens and attitudes toward deprescribing in older inpatients. A recent study that investigated the association of three items of rPATD with MRCI in 42 older adults with HIV found no significant association [37].

Our study found that increased frailty risk was associated with lower odds of willingness to deprescribe. Patients with intermediate hospital frailty risk score had 65% lower odds of willingness to deprescribe compared to the low-risk group. To our knowledge, no previous studies have investigated the association of hospital frailty risk score with willingness to deprescribe in older inpatients. An Australian study on older inpatients’ attitudes toward deprescribing statins found that frail patients (measured by the Reported Edmonton Frail Scale) were less willing to stop a statin compared to robust individuals [24]. Other studies that have examined the association of frailty (based on other criteria, not HFRS) and medications have found an increased risk of prefrailty and frailty in patients with polypharmacy [38–40]. A recent systematic review of 37 studies showed that 47% and 59% of prefrail and frail patients had exposure to polypharmacy, respectively [38]. In our study, 55% of patients with low risk and 35% of those with intermediate hospital frailty risk scores had polypharmacy. However, two-thirds of patients with intermediate frailty risk were taking < 5 medications and this could be one possible reason for the lower odds of willingness to deprescribe observed in groups with intermediate frailty risk. Frailty is associated with increased exposure to polypharmacy and anticholinergic burden [1, 41] and potentially inappropriate medications (PIMs) [42] and, this increases the risk of adverse events such as falls and hospitalization [1, 43]. Studies examining the association between frailty and medication may be hampered by the variability in the definitions, as well as measures used to assess frailty. Furthermore, frail older adults are often not included in clinical trials [44] and thus, the true effect of medications (i.e. net benefit or risk) may not be noticeable compared to robust individuals. To date, evidence on a causal relationship between frailty and medications is inconclusive [39]. There is limited high quality data on the association of frailty with attitudes toward deprescribing in older inpatients. Hospital frailty risk score is relatively a new measure and has not been widely used in the inpatient setting compared to other measures of frailty particularly, in the context of medication use and deprescribing. Therefore, the complex interplay between frailty and polypharmacy and the influence of this on patient’s willingness to deprescribe in an acute setting needs further investigation.

It is interesting to find that history of falls was associated with lower odds of perceived burden. The association between self-reported burden and falls has not been well investigated in previous studies. However, there is strong evidence on the association between medication burden (objectively measured as ≥ 5 medications) and the risk of falling [40]. In our study, out of 43 (19.4%) patients who reported that medicines are a burden to them (item 4 of the burden domain), one-third had a history of falls in the past month. Likewise, out of 199 patients with polypharmacy, 20% reported a high perceived burden, and 43.1% had a history of falls. This may imply that patients with a history of falls may not necessarily view that their medications as burdensome and show willingness to deprescribe, yet they could be on polypharmacy or medications that may increase the risk of falls and rehospitalisation. A recent US study of 422 older adults with dementia found that patients with a history of falls are less likely to endorse willingness to deprescribe compared to those with no history [34]. Due to the lack of robust data on the association of falls with self-reported burden and willingness to deprescribe, clinicians need to be alert about the possibility of fall-risk-inducing medications particularly, in patients taking a higher number of medications even if they report low perceived burden. The present study did not investigate different classes of medications associated with history of falls however, a recent systematic review showed that fall-risk-increasing drugs (FRIDs) are common in older adults and the prevalence ranges from 65 to 93% with antidepressants being the most common FRID [45].

Consistent with previous studies, this study found that the oldest old age group had lower odds of perceived burden and desire to involve in decision-making and were less wanting to try stopping their medications to see how they feel without them. A study of 1981 older adults in the US reported that older adults aged 85 years and older had lower odds of wanting to reduce their medications [33]. Contrary to this, other studies reported no significant association between age and attitudes toward deprescribing [31]. A systematic review and meta-synthesis on medication burden revealed that some patients feel overwhelmed by the number of medications they take whilst others perceive their medications as important for their health irrespective of the number [21]. While the observed association between age and attitudes toward deprescribing in our study cohort needs further investigation, this equally, indicates that there is a need to educate older inpatients about their medications, encourage them to share concerns they may have about medication burden, and more importantly triage them for deprescribing intervention. Our findings on participants’ demographic data also showed that female patients had less interest in being involved in decision-making compared to males. Some studies have shown that older men often tend to report dissatisfaction with their medications [36] and are more likely to want to reduce the number of medications [33] compared to older women. Other studies reported no association between sex and attitudes toward deprescribing [46]. Hence, clinicians should not dismiss deprescribing opportunities in subgroup of inpatients who may be less interested in being involved in decision-making or dissatisfied with their medications [31, 32].

### Limitations

There are several limitations to this study. Although all items of the rPATD tool were used in the descriptive summary, only six selected items were included in regression analysis and, therefore, the association of other items with identified participant characteristics were not investigated. As the rPATD tool is a generic measure, it is unclear which specific medication or class of medications the participants have in mind when responding to the questionnaire. Furthermore, the rPATD tool has not been validated for use in the New Zealand population. We did not investigate the views of carers in our study as we included only patients who were able to self-manage their medications and were interested in patients’ capacity in making independent decisions without being dependent on carers. We believe that the views of carers or patients who are dependent on carers may differ from the results observed in this study.

The study enrolled participants from general medicine and geriatric medicine services thus, the findings may not be applicable to other inpatient specialities and patients receiving care in outpatient clinics. The characteristics of our sample may also not reflect the socio-demographics of the rest of the country; notably there was under representation of certain ethnic groups such as Māori, Pacifika in our sample. Although a comparison of attitudes toward deprescribing by ethnicity was not the focus of the present study, the findings on ethnicity data should be interpreted with caution.

We have adjusted for factors that influence individuals’ attitudes toward deprescribing such as number of medications, the complexity of the regimen, comorbidities, and frailty however, we have not explored the class and clinical appropriateness of the medications. The presence of potentially inappropriate medications may influence attitudes toward deprescribing. Future research should take the specific class and appropriateness of medications into consideration in addition to other factors identified in this study.

## Conclusion

Most older inpatients had a high desire to be involved in decision-making about their medications and were willing to stop one or more medications if proposed by their prescriber. Medication complexity, frailty status, sex and age of the patient influence attitudes toward deprescribing and thus, should be taken into consideration when making deprescribing decisions. Further research is needed to investigate the relationship between frailty and the complexity of medication regimens.

## Data Availability

The datasets generated during this study are not publicly available due to privacy/ethical restrictions, but anonymised data are available from the corresponding author upon reasonable request.

## Abbreviations

**AOR**: Adjusted odds ratio
**ADEs**: Adverse drug events
**CCI**: Charlson comorbidity index
**CIs**: Confidence intervals
**COR**: Crude odds ratio
**FRIDs**: Fall risk inducing drugs
**HFRS**: Hospital frailty risk score
**IQR**: Interquartile range
**MRCI**: Medication regimen complexity index
**PIMs**: Potentially inappropriate medications
**SD**: Standard deviation
**rPATD**: Revised patient Attitude toward Deprescribing
